# What Is the State-of-the-Art in Clinical Trials on Vaccine Hesitancy 2015–2020?

**DOI:** 10.3390/vaccines9040348

**Published:** 2021-04-05

**Authors:** Carla Pires

**Affiliations:** Research Center for Biosciences and Health Technologies, CBIOS-Universidade Lusófona de Humanidades e Tecnologias-Escola de Ciências e Tecnologias da Saúde, Campo Grande 376, 1740-024 Lisboa, Portugal; p5558@ulusofona.pt

**Keywords:** vaccine hesitancy, vaccination, vaccine refusal, hesitancy determinants, immunization, randomized controlled trial, clinical trial

## Abstract

Background: Vaccine hesitancy is related to a delay in acceptance or refusal of vaccination. Aim: to perform a systematic review of clinical trials on vaccine hesitancy (2015–2020). Methods: a systematic review following the Preferred Reporting Items for Systematic Reviews and Meta-Analyses criteria (PRISMA). Five databases were screened—PubMed, Cochrane Library, DOAJ, SciELO and b-on—which comprise multiple resources. Keywords: “Vaccine hesitancy” and (“randomized controlled trial” or “clinical trial”). Inclusion criteria: trials about “vaccine hesitancy” enrolling patients and/or health professionals (2015–2020). Exclusion criteria: studies about other topics, repeated and qualitative studies, reviews and papers written in languages other than English, Portuguese, French or Spanish. Results: a total of 35 trials out of 90 were selected (19 PubMed, 14 Cochrane Library, 0 DOAJ, 0 SciELO and 2 b-on). Selected trials were classified into five topics: children/pediatric (n = 5); online or electronic information (n = 5); vaccination against a specific disease (n = 15) (e.g., influenza or COVID-2019); miscellaneous (n = 4); and educational strategies (n = 6). Conclusion: the provision of online or electronic information (e.g., through virtual reality, social websites of experts, or apps), communication-based interventions and training of health professionals, residents or subjects seemed to improve vaccine hesitancy.

## 1. Introduction

Citizens who are vaccine hesitant show a variable degree of indecision about getting a specific vaccine or about vaccination in general [1]. According to the SAGE Working Group on Vaccine Hesitancy, the term vaccine hesitancy refers to a delay in acceptance or refusal of vaccination, despite availability of vaccination services [2]. Principally, parents’ attitudes were classified into five groups, as follows: unquestioning acceptors, cautious acceptors, hesitant parents, late or selective acceptors, and parents who refuse all vaccines [3]. Vaccine hesitancy factors may be classified as follows: individuals’ perceived risk of vaccine-preventable diseases (e.g., a limited knowledge of vaccine-preventable diseases); socio-demographic variables (e.g., number of years of schooling); complacency (e.g., vaccination is not required, since knowledge of the risks of vaccine-preventable diseases is low); convenience (e.g., physical availability, affordability, price and/or accessibility of vaccines); confidence (e.g., effectiveness and safety of vaccines or competence of health services); political motivations (e.g., vaccine policies/programs); or characteristics of the healthcare system (e.g., paid/free vaccines) [1,2,3,4].

Vaccine hesitant individuals are represented by heterogeneous groups (personal, family and/or community). These groups may be influenced by different intrinsic and extrinsic elements (e.g., individual, contextual, and/or group factors) [2,3,4]. Among the individual and group factors for vaccine hesitancy are the subjects’ experience with vaccination (e.g., pain), beliefs, attitudes about health and prevention, knowledge/awareness, trust of health systems and providers, perceived risk/benefit, immunization as a social norm, scientific evidence, perception about a new vaccine, a new formulation or a new recommendation, mode of administration or design of vaccination program. Additionally, among the contextual factors that may influence vaccine hesitancy are media communication/information, anti-or pro-vaccination groups/lobbies, immunization programs or immunization policies, health regulation, religion, culture, gender, socio-economic status, country history, geography (e.g., geographic barriers), or opinions about the pharmaceutical industry [2].

Vaccine hesitancy relates to low vaccination rates (e.g., parents who tend to be more vaccine hesitant are less likely to vaccinate their children). In general, the more hesitant parents are older and more educated. These parents show more fear of drug adverse reactions, describe past negative experiences with health services, report fear of developing a pathology (e.g., autism) or report the undesirable histories of family and friends [2,5]. Parents seem to be more vaccine hesitant in relation to ‘new’ vaccines (e.g., HPV, meningococcal, or pneumococcal) in comparison to older ones (e.g., Measles–Mumps–Rubella, Diphtheria–Tetanus–Pertussis) [5].

In low- and middle-income countries, parent’s education, immunization reminder cards, household incentives, home visits or integration of immunization with other services are among the most frequent interventions to improve childhood immunization coverage [6]. Vaccine hesitancy should be studied in diverse settings since this condition is multifactorial and context-specific [4]. For instance, parent’s beliefs and attitudes [7]; online or electronic information [8]; type of vaccine-preventable disease (e.g., human papillomavirus infection or influenza) [9,10]; pain or fear of needles [11,12] or training/education on vaccination [13] may influence vaccine hesitancy [4,7,8,9,10,11,12,13].

Studies about vaccine hesitancy seem to be especially relevant due to the need to ensure high vaccination rates to achieve herd immunity during the COVID-2019 pandemic [14,15,16]. A study enrolling 1941 healthcare professionals and participants of the general Israeli population was conducted through the administration of an anonymous questionnaire. According to the findings of this study, healthcare workers taking care of COVID-2019 positive patients or subjects perceived as being at high risk of disease were more likely to self-report compliance to COVID-2019 vaccination than parents, nurses, and medical workers not taking care of SARS-CoV-2 positive patients (i.e., the latter manifested higher levels of vaccine hesitancy) [14]. In a survey carried out in France (n = 3259 participants) (26 March–20 April 2020), the determined factors associated with the acceptance of COVID-2019 vaccine were older age, male gender, fear about COVID-2019, being a healthcare professional and the perceived risk of this disease [15]. Significant predictors of COVID-2019 vaccine uptake intentions were as follows: higher education, having insurance, scoring high on subjective norms (e.g., “People who are important to me would approve of me getting a COVID-2019 vaccination”), a positive attitude toward the vaccine, a high perceived susceptibility to COVID-2019, high perceived benefits of the vaccine, scoring low on barriers to the vaccine (e.g., “The development of a COVID-2019 vaccine is too rushed to properly test its safety,” and “I am concerned about the side effects of a future COVID-2019 vaccination”), and scoring high on self-efficacy (n = 788 participants, survey in USA) [16].

Thus, the aim of this study was to perform a systematic review of the clinical trials (e.g., randomized and controlled clinical trials) on vaccine hesitancy between 2015–2020. Qualitative and quantitative evidence of the selected studies were collected with regard to study objectives, number of participants, methods and results, and conclusions.

The following research question was defined: What is the evidence from clinical trials on subject´s vaccine hesitancy between 2015–2020?

## 2. Materials and Methods

The Preferred Reporting Items for Systematic Reviews and Meta-Analyses (PRISMA) and PICOS (P: Participants; I: Intervention; C: Comparisons; O: Outcomes; S: Study design) were followed (see Section 2.4.1. PICOS criteria for the inclusion of studies). PRISMA orientations were applied to ensure a transparent reporting of evidence. PICOS was followed to define the research question, study aim and the criteria for the inclusion of studies. A protocol following the PRISMA checklist was previously developed. This protocol is not publicly available because its full content is presented and explained here [17].

### 2.1. Previous Identified Systematic Eeviews

#### 2.1.1. Cochrane Library

Between 2015–2020, only one systematic review has been identified in the Cochrane Library with the selected keywords and Boolean operators “vaccine hesitancy” and (“randomized controlled trial” or “clinical trial”) [6,18]. However, this systematic review was about other topic [6]. According to study findings, face to face interventions to inform/educate parents about childhood vaccination had a restricted impact on the status of immunization, and on parents’ knowledge or understanding of vaccination. Although parents’ intention to vaccinate their children has been defined as a secondary outcome, authors reported insufficient evidence on the impact of training parents/caregivers on children’s vaccination [6].

#### 2.1.2. PubMed

The criteria applied to identify related-systematic reviews were as follows: “vaccine hesitancy” (search term), with the automatic filler for “systematic reviews” of PubMed activated (8 February 2021). Among the 29 identified systematic reviews about “vaccine hesitancy” published in the last 5 years in PubMed (8 February 2021), none were exclusively about intervention studies concerning subjects´ vaccine hesitancy (i.e., a systematic review of trials, with the aim of studying the impact of a certain intervention or a certain variable on subjects´ vaccine hesitancy). These findings reinforce the relevance of the present systematic review.

However, a secondary search in PubMed found a systematic review about the present topic, which was that of Jarrett et al. (2015) [19]. Jarrett et al. searched for both grey and peer-reviewed literature. For peer-reviewed literature, the following databases were searched (January 2007–October 2013): Medline, Embase, PsychInfo, Cochrane, CINAHL Plus, Web of Science, LILACS, Africa-Wide Information; IBSS; and IMEMR, i.e., around 6 years and 10 databases. The applied keywords were “strateg *”, “intervent *”, “campaign”, “evaluation”, “approach”, or “program*” (six keywords). Among the inclusion criteria were studies that “described or evaluated an intervention addressing hesitancy and reported a measure of the primary outcome, i.e., indicating a change in vaccination uptake or the secondary outcome, i.e., indicating a change in knowledge/awareness and/or attitudes.” Overall, 166 (peer reviewed) and 15 (grey literature) items were selected by Jarrett et al. [19].

Importantly, the search period of the present systematic review (2015–2020) is not covered in the systematic review by Jarret et al. (2007–2013) [19]. Additionally, grey literature was not considered in the present systematic review to avoid potential study imprecisions or errors, since peer reviewed works are expected to be more accurate. The timeframe (measured in number of years) is very similar between Jarret et al. (n = 6 years) and the present systematic review (n = 5 years). More keywords were applied in the systematic review of Jarret et al. [19] (see *4.9. Potential Study Limitations of the Present Systematic Review*).

The limited number of identified systematic reviews (Cochrane Library and PubMed) that were published in the last 5 years exclusively about the revision of interventions on subjects´ vaccine hesitance reinforces the relevance of the present systematic review.

### 2.2. Screened Databases/Resources and Dates of Data Collection

This systematic review was carried out by one researcher (the study author) who double-checked all searches and study findings. The period between 2015–2020 was defined to identify recent and updated papers/works on the present topic, which is in line with the methodologies of other similar systematic reviews [4,19].

#### 2.2.1. Screened Databases/Resources

Five databases have been conveniently screened (PubMed, Cochrane Library, DOAJ, SciELO, and b-on) [18,20,21,22,23], because these databases comprise a significant number of indexed papers, works, chapters, journals, and other publication sources. Their main characteristics are presented, as follows:

- PubMed is maintained by the U.S. National Library of Medicine, USA and includes over 30 million citations for biomedical literature e.g., MEDLINE, life science journals, and/or online books [20].
- Cochrane Library is a database of accessible systematic reviews and other synthesized research evidence, namely the Cochrane Database of Systematic Reviews (4–12 issues, since 2003) and Cochrane Central Register of Controlled Trials (CENTRAL) (e.g., CENTRAL is updated source of reports of randomized and quasi-randomized controlled trials, namely from PubMed and Embase-Biomedical database from Elsevier). The mission of Cochrane Library is to promote evidence-informed health decision-making by producing high-quality, relevant, accessible systematic reviews, and other synthesized research evidence [18].
- DOAJ is an online directory that provides access to papers form high quality, open access, peer-reviewed journals (80 languages; 123 countries; 15,678 journals and 5,516,249 articles records on 2 January 2020 [21].
- SciELO is a database comprising a high-quality collection of Brazilian scientific journals (381 journals: 427,662 documents on 2 January 2019) [22].
- b-on provides unlimited and permanent access to research (thousands of scientific journals and online e-books from some of the most important content providers). It started on March 2004, brings together different institutions, such as higher education, scientific research and technological development, hospitals [23]. Importantly, the following collections are available in b-on: Academic Search Complete, Annual reviews, Current Contents (ISI), Elsevier, Essential Science Indicators, IEEE, EBESCO (LISTA), Nature, Sage, Springer, Taylor and Francis, Web of Science and Wiley, among many others (please see https://www.b-on.pt/colecoes, accessed on 5 April 2021).

Some databases were not purposively included. For instance, Medline is indexed in PubMed [20], and CINAHL includes many of the same features as PubMed [24]. The number of searched databases was considered adequate in line with the design of previous systematic reviews [4,19] and the PRISMA requisites for conducting systematic reviews [17], since an expressive number of databases were included in the present study.

#### 2.2.2. Dates of Data Collection

Studies were identified in PubMed, Cochrane Library, DOAJ, and SciELO on 28 December 2020, and in b-on on 28 December 2020 and 29 December 2020, with the previously defined keywords. Prints of the lists of outputs were produced for each database. No new outputs were detected on 1 January 2021.

### 2.3. Keywords

The screened keywords and Boolean operators were “vaccine hesitancy” and (“randomized controlled trial” or “clinical trial”). These keywords were conveniently selected to restrict the search about “vaccine hesitancy”. The choice of “randomized controlled trial” or “clinical trial” aimed to specifically identify intervention studies about “vaccine hesitancy”, with the involvement of patients or health professionals. As far as the author knows, this is the first systematic review specifically applying these keywords (see *2.1. Previous Identified Systematic Reviews*).

A systematic review using the keywords “vaccine hesitancy” or “vaccine refusal” plus diverse types of study design was found. This systematic review aimed to summarize the evidence surrounding childhood vaccine hesitancy from the perspective of parents, but qualitative research was included [25].

#### 2.3.1. Randomized Controlled Trial or Clinical Trial

These two keywords were selected to target the selection of potentially relevant studies. Evaluation of the impact of a certain intervention or the impact of a certain variable on vaccine hesitancy was considered more likely in studies that included these keywords, i.e., “randomized controlled trial” or “clinical trial” and “vaccine hesitancy” [25].

#### 2.3.2. Vaccine Hesitancy

“Vaccine hesitancy” was selected because it is an emergent keyword in article databases. For instance, a search for “vaccine hesitancy” (8 February 2021) retrieved 1079 results in PubMed, with almost all results being available only after 2015. A previous systematic review by Jarret et al. in 2015, which looked at strategies for addressing vaccine hesitancy, reported that “the term ‘hesitancy’ or ‘hesitant’ with reference to vaccines/vaccination was only detected in 0.4% of the studies across all the reviewed literature (1208 articles)” [19]. This finding (0.4% of the studies) is no more applicable nowadays, since “vaccine hesitancy” retrieved 1079 outputs in PubMed (8 February 2021), which corresponds to almost the same number of studies identified by Jarret et al. in 2015 (n = 1208) [19].

#### 2.3.3. Vaccination Refusal

The MeSH term “vaccination refusal” was created in 2017 (https://www.ncbi.nlm.nih.gov/mesh/?term=vaccine+refusal, accessed on 5 April 2021). The initial motive for not selecting “vaccination refusal” was because this expression is more restrictive than “vaccine hesitancy”. In addition, a search in PubMed using “vaccination refusal” on 8 February 2021, retrieved 508 results (almost all available after 2015), with 186 out of the 508 outputs also comprising the word “hesitancy”. From these 508 results, only 5 outputs were from clinical trials or randomized controlled trials (the automatic classification about the article type: “clinical trial” and “randomized controlled trial” were selected in the automatic search options of PubMed). These five studies were classified as follows: one systematic review out of the study period, three systematic reviews already included in the present work, and one study protocol. Thus, “vaccination refusal” was not included as a keyword in the present work.

### 2.4. Inclusion and Exclusion Criteria

#### 2.4.1. PICOS Criteria for the Inclusion of Studies

All clinical trials (S: Study design) (e.g., randomized and controlled clinical trials) (C: Comparison), about the impact of a certain intervention or the influence of a certain variable (I: Intervention) on “vaccine hesitancy” (O: Outcome) enrolling patients and/or health professionals (P: Participants), which have been published between 2015–2020 (last five years) were included in the present systematic review. This period was defined to identify recent and updated papers/works on this topic, which is in line with the timeframes of other systematic reviews about similar topics [4,19].

#### 2.4.2. Exclusion Criteria

Works/papers that were outside of the defined period (i.e., 2015–2020), repeated studies, reviews and qualitative research or any study not published as a “randomized controlled trial” or a “clinical trial”, grey literature, and papers written in languages other than English, Portuguese, French, or Spanish were excluded, the latter being due to economic restrictions related to translation costs. As already explained, grey literature was excluded to avoid potential mistakes or imprecisions, because these documents are usually not peer reviewed.

## 3. Results

### 3.1. Selected Studies

**Figure 1:**
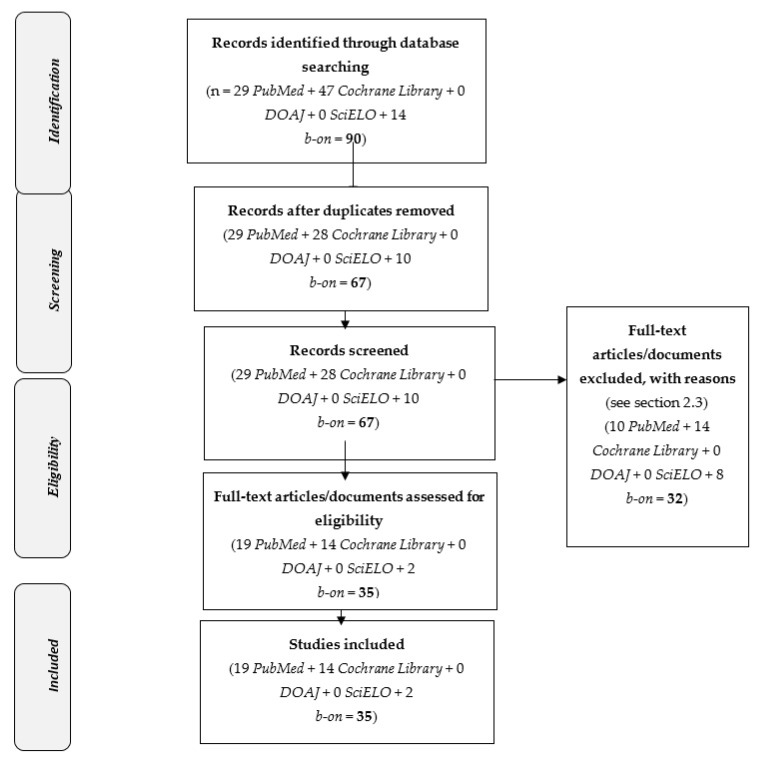
PRISMA 2009 Flow Diagram [17]: Selected studies on “vaccine hesitancy” and (“Randomized Controlled Trial” or “clinical trial”) (2015–2020).

Overall, 35 full-text articles/documents were assessed for eligibility: 19 PubMed, 14 Cochrane Library, 0 DOAJ, 0 SciELO and 2 b-on (Figure 1).

### 3.2. Main Findings of Selected Studies

The main findings of the 35 selected studies are presented in Table 1, which is organized by author(s), year of publication, country, database, objective(s), number of participants, methods and results, and conclusions. These studies accounted for at least 2023 health professionals or residents and 59,467 subjects.

The selected studies were organized into five topics: children/pediatric (n = 5); online or electronic information (n = 5); vaccination against a specific disease: human papillomavirus infection (n = 9), influenza (n = 3), diphtheria tetanus toxoid and pertussis (n = 2) and COVID-2019 (n = 1); miscellaneous: extrinsic factors (n = 4); and educational strategies (n = 6) (Table 1). The selected studies were grouped into just one of these five topics for practical reasons and to avoid the duplication of information. Papers are presented from the most recent publication date to the oldest date per each topic/section.

## 4. Discussion

This section is organized according to the number and type of topics identified in the Section 4.1, Section 4.2, Section 4.3, Section 4.4 and Section 4.5 (i.e., children/pediatric, online, or electronic information, vaccination against a specific disease, miscellaneous: extrinsic factors and educational strategies, respectively) plus four additional Section 4.6, Section 4.7, Section 4.8 and Section 4.9 which relate to study limitations and studies biases, future research directions, study strengths, and potential study limitations of the present systematic review, respectively.

The evidence collected by Jarrett et al. in 2015 was essentially focused on influenza, human papillomavirus, and pediatric vaccines (58% of the selected studies) [19]. The present systematic review comprises a wider diversity of studied topics (i.e., children/pediatric, online, or electronic information, vaccination against a specific disease, miscellaneous: extrinsic factors and educational strategies). Jarrett et al. opted to discuss the selected evidence per general topics (i.e., dialogue-based interventions, non-financial incentives, and reminder-recall interventions). This structure contrast with the organization of the present systematic review where evidence is discussed for each detailed topic [19]. In some cases, organizational differences may have limited the comparison of data/findings between the two systematic reviews.

Thirty-five studies have been selected in the present systematic review, which is in line with the number of studies identified in other systematic reviews about related topics. For instance:

- n = 27 studies were selected in a systematic review that aimed to summarize evidence surrounding childhood vaccine hesitancy from the perspective of parents. The main conclusion of this systematic review was that healthcare professionals should take the caregivers’ “desire to do what they feel right for the child” into consideration [25].
- n = 43 studies were selected in a systematic review that aimed to evaluate differences by individual socioeconomic status in terms of the uptake of publicly funded childhood vaccines and in cognitive determinants (beliefs, attitudes) of parental decisions about vaccinating their children in developed countries with programs addressing major financial barriers to vaccination access. This systematic review concluded that mandatory and recommended vaccines should be provided 100% free of charge and the administration of vaccines reimbursed, since barriers to vaccination access persist, and tailored interventions are recommended for vaccine-hesitant parents [54].
- n = 11 studies were selected in a systematic review that aimed to provide quantitative comparative data on any measure of vaccine uptake. According to the findings of this systematic review: email communication seems to increase vaccine uptake when compared with no intervention [55].

However, a larger number of peer reviewed studies (n = 166) were selected in the systematic review of Jarrett et al. (2015) [19] (the only identified systematic review about the same topic—i.e., exclusively, experimental studies about vaccine hesitancy between 2007 and 2013. The application of a higher number of different and general keywords (i.e., “strateg *”, “intervent *”, “campaign”, “evaluation”, “approach”, or “program *”) in this previous systematic review compared to the present review (i.e., “vaccine hesitancy” and (“randomized controlled trial” or “clinical trial”)) may explain the higher number of studies selected by Jarrett et al. (n = 166 in Jarret et al. (2015) vs. n = 35 in the present systematic review), although further research would be necessary to confirm this hypothesis.

Among other objectives, the systematic review of Jarret et al. aimed to evaluate vaccine hesitancy across diverse global contexts. This evaluation was not specifically covered in the present work, which may explain the superior number and type of keywords applied by Jarrett et al. [19]. The keywords of the present study seem to be more precise and specific, regarding the topic of vaccine hesitancy. Additionally, the number of published papers containing the expression “vaccine hesitancy” has exponentially increased since 2015, according to *PubMed* metrics:(n = 1081 results; 9 February 2021, almost all available after 2015, which corresponds to almost the same number of peer reviewed articles by full text (n = 1149) in Jarrett et al. (2015) [19].

### 4.1. Children/Pediatric

The number of trials involving parents and/or health professionals about vaccine hesitancy was scarce and involved a limited number of parents and/or health professionals. Five randomized controlled trials about this topic have been identified: three about parents’ vaccine hesitancy, one about additive pain interventions, and one involving medical students and pediatric residents, who were questioned about diverse scenarios [7,11,26,27,28]. Importantly, evaluation of vaccine hesitancy with Parent Attitudes About Childhood Vaccines Survey (PACV) not significantly impacted the % of days of children under-immunized [7]. As expected, vaccine hesitancy seems to diminish as parents’ experience with vaccines increase after childbirth [26].

Acute pain and distress during vaccination in infants may contribute to increase the level of parents´ dissatisfaction with vaccination and vaccine hesitancy. The administration of topical lidocaine seems to be beneficial to control pain and avoid parents’ vaccine hesitancy [28]. Furthermore, nonpharmacological methods, such as breastfeeding or 24% sucrose solution reduced children vaccination pain in infants up to 6 months. Positively, these nonpharmacological interventions may reduce parents’ vaccine hesitancy [11]. Medical students and pediatric residents reported a less favorable opinion about potential vaccine hesitant parents, with a potential negative impact on the conversations between residents and patients about vaccination [27].

Research findings do not seem to be extended to the general population since the evaluated studies were neither representative nor multicentric (e.g., national research with the application of formulas to calculate representative samples sizes were not found) [7,11,26,27]. Other variables may be investigated regarding the impact on vaccination rates, such as caregivers’ or health professionals’ sociodemographic features, social condition, previous experience with vaccination (e.g., pain or mode of administration), fear of needles or adverse drug reactions, opinion about health professionals or the health system, type of models to train residents or health professionals (e.g., pediatricians), etc.

Other systematic reviews about parents’ vaccine hesitancy (e.g., qualitative research), also address the importance of health professionals consider caregivers’ opinions and desires through:

- Tailoring the information to the target audience.
- Understanding parent’s hesitancy, and the specific context (e.g., management of misinformation about vaccination).
- Presenting vaccination as the default approach (e.g., early in pregnancy in prenatal appointments and the first postnatal appointment); both social and individual responsibility should be communicated.
- Using technology to promote vaccination.
- Improving parents’ vaccine literacy and critical thinking skills (e.g., vaccine education materials) [25,56].

Healthcare professionals were identified as the most trustworthy messengers, who should provide balanced information (e.g., risks vs. benefits). Among the communication techniques were the storytelling, emotive anecdotes, or other persuasive messaging strategies [56]. However, the evidence about the impact of face-to-face information between health professionals and caregivers on children’s vaccination status seems to be low to moderate. Thus, the use of both pharmacological and non-pharmacological interventions during children vaccination may be especially relevant to reduce parents’ vaccine hesitancy [11,28].

### 4.2. Online or Electronic Information

Validated, usable, comprehensive, rigorous, and appellative online or electronic resources, such as apps, websites, internet-based interventions/platforms, games, or social media applications are potentially useful to diffuse correct and intelligible information about vaccination [8,29,30,31,32]. For instance, a previous examination of literature reported that parents frequently seek for vaccine information in online sources, which frequently disseminate misinformation about vaccine risks [57].

Only, five trials about the impact of online or electronic information on parents´ vaccine hesitancy have been identified in the present systematic review [8,29,30,31,32]. In general, online, or electronic information may produce a positive impact on subjects’ vaccine hesitant, as follows:

- Virtual reality through a head-mounted display unit promoted participant’s understanding of key immunization concepts and their integration in the story [8].
- An expert moderated vaccine social media website constituted a suitable platform for parents to collect accurate vaccine information, express vaccine concerns, and/or ask questions to vaccine experts; this site was developed for parents of children 24 months of age or younger [29].
- The use of an app produced positive outcomes (e.g., provision of appropriated vaccine information) [30].
- Among vaccine hesitant pregnant women, an Internet-based intervention/platform positively enhanced vaccine hesitant parent’s attitudes on vaccines [31]; and
- During pregnancy, parental vaccine behaviors may be positively influenced by a web-based vaccine information with social media applications [32].

Besides the lack of representativeness, an increased rate of vaccination after the administration of online or electronic information was not demonstrated in the selected studies [8,29,30,31,32]. Thus, future studies should be longitudinal and prospective aiming at evaluating the impact of providing online information on both subjects’ vaccine hesitancy and vaccination rates.

### 4.3. Vaccination Against a Specific Disease

The number of trials about vaccine hesitancy on specific diseases was also restricted (e.g., Human papillomavirus infection; influenza; diphtheria tetanus toxoid and pertussis and COVID-2019) [9,10,33,34,35,36,37,38,39,40,41,42,43,44].

#### 4.3.1. Human Papillomavirus Infection

Nine studies about HPV vaccination were identified [9,12,33,34,35,36,37,38,39]. The main findings of these studies:

- Preadolescent fear of needles was a negative predictor of subsequent HPV vaccine uptake. This fear may be reduced by avoiding same-day preschool injections (4–6 years); i.e., the administration of two immunizations on the same day should be preferably avoided [12].
- A significant number of Latin immigrant mothers of daughters (9–12 years) (around one-third of participants) was HPV vaccine hesitant. Thus, besides recommending HPV vaccination, health professionals should raise awareness on additional HPV complications e.g., HPV and cervical cancer or reinforcing daughters’ perceived risk of HPV infection [9].
- A multi-component communication-based intervention improved healthcare provider’s communication about HPV vaccination (11–12 years children), with improved HPV vaccination rates [33].
- Tailored messages (e.g., video) addressing all concerns improved HPV vaccination produced positive outputs (e.g., improvement of vaccination intent) [34,36].
- A provider’s ‘very strong’ recommendation on adolescent vaccination was associated with a greater perceived urgency for getting vaccinated, greater trust in the information received, decreased vaccine hesitancy, and increased vaccine receipt [35].
- Motivational interviews facilitated dialogues between health professionals (e.g., nurse practitioner or physician assistant) and vaccine-hesitant parents, with the improvement of HVP vaccine acceptance [37].
- Written information, reminders, or multicomponent interventions (e.g., HPV fact sheets or related images) augmented HPV vaccination [38,39].

Communication-based interventions between health professionals and parents or adolescents assisted the adherence to HPV vaccination and/or the vaccination intention among adolescents. Thus, communication about HPV vaccination between caregivers and health professionals should be carefully tailored and conducted.

Particularly, the quantity and quality of information available about HPV vaccination. For instance, subjects’ mistrust on health authorities, healthcare workers, or new vaccines or concerns on side effects were reported as the most relevant determinants of HPV vaccine hesitancy in Europe (the region with the least confidence in vaccination at a global level) [58].

#### 4.3.2. Influenza

Only two controlled trials with hesitant participants on getting influenza vaccination were identified [10,40]. Positively, one study was multicentric (36 primary care practices in 24 states) [10], although none of the identified studies was representative at national level [10,40]. Pediatricians should promote vaccination after the administration of the first dose of influenza vaccine since many caregivers remain vaccine hesitant even after the administration of the first dose. Additionally, health professionals should address inaccurate influenza beliefs for the reason that parents showed misperceptions about influenza disease and vaccination [10]. Participant’s perceptions and attitudes about vaccination were not modified by online messages (i.e., original participants’ perceptions and attitudes were dominant), with participants not disseminating these messages [40].

Tailored communication interventions by health professionals were relevant to promote vaccine uptake and avoid vaccine hesitancy (e.g., caregivers of children who received the first of two doses of influenza vaccine). Although, contrary to what would be expectable online messages were not sufficient impactful to change participants’ perceptions and attitudes toward flu vaccination and/or to facilitate the dissemination of these messages [10,40].

Once more, the number of selected studies about this topic was limited. Thus, further studies about hesitant subjects, regarding influenza vaccination are recommended. For instance, studies about the possible impact of communication studies (e.g., communication interactions between health professionals and caregivers or other adults, such as geriatric, diabetic, or oncologic patients), social networks (e.g., Facebook or Instagram), or other media (e.g., TV, journals, and magazines) on vaccine hesitancy or studies to evaluate how subjects’ health literacy and years of schooling is related to vaccine hesitancy.

In addition, future communication interventions, including the diffusion of online messages should address the most frequently reported barriers to influenza vaccination, such as subjects´ decreased perceived effectiveness of the vaccine, a lack of trust in health authorities, safety concerns, low perceived severity/risk of the disease, lack of recommendations by health workers or a limited number of interactions with health services. Other enablers of vaccine uptake should also be considered when developing online messages, such as subjects’ high perceived utility of vaccination, cues to action, or previous influenza vaccinations [59].

#### 4.3.3. Diphtheria Tetanus Toxoid and Pertussis

As expected, vaccination informative websites were potentially relevant to increase diphtheria tetanus toxoid and pertussis (Tdap) and influenza vaccine uptake (e.g., website with vaccine information and interactive social media components), among pregnant women. However, outputs only were statistically significant for influenza vaccination in this study [42]. Furthermore, immunization campaigns in health camps may benefit from symbolic necklaces to raise the attention on Tdap vaccination [43].

The number of identified trials about Tdap vaccine hesitancy was scarce, which is aligned with the findings of the systematic review of Jarrett al. (2015) [19]. Jarrett al. (2015) reported that reminder–recall interventions increased DTP3 vaccination (one study Pakistan), social mobilization among parents produced a positive effect on DTP1 and DTP3 (evidence varied between moderate to low, one study Pakistan and one study Nigeria), communication tool-based training for health care workers had a positive effect for DTP3 (one study Pakistan) and an information-based training for healthcare workers increased DTP3 vaccination (one study Turkey) (findings for low-income countries) [19].

Further research about vaccine hesitancy on diphtheria vaccination is recommended, since maintenance of immunization against this disease in developed and developing countries is necessary. The majority of adults over 50 may loss immunity against diphtheria infection, which reinforces the need of immunity maintenance against diphtheria by boosters in adults (e.g., an outbreak of this infection was reported in Eastern European countries in the 1990s) [60].

#### 4.3.4. COVID-2019

Just one review about COVID-2019 vaccination was selected. A straighter collaboration between health professionals and certain less favored communities (e.g., HIV-positive Black Americans) was recommended in this study, aiming at developing and implementing personalized strategies to promote COVID-2019 vaccination and treatment uptake [44]. Similar study findings are supported by other study: White Americans consistently expressed higher receptivity to COVID-2019 vaccination, in opposition to Black Americans, who showed more mistrust and lower confidence in this vaccine [61].

Additional studies about COVID-2019 vaccine hesitancy are recommended, because of the urgent need of massive vaccination to achieve herd immunity against SARS-CoV-2, the reported high levels of COVID-2019 vaccine hesitant subjects, and the likely regional differences between countries [14,15,16]. Future research should be tailored per country since the motives to avoid COVID-2019 are diverse. For instance, the most common motives of hesitation/refusal of COVID-2019 vaccine are lower income, uninsured, living in rural areas or larger households. Among the most common motives for hesitation/refusal or lower vaccination acceptance are the fear of side effects, vaccine safety, and vaccine effectiveness, the belief that vaccines are unnecessary, inadequate or mistrust information, uncertain about the duration of immunity, and a general anti-vaccine stand [61].

### 4.4. Miscellaneous: Extrinsic Factors

#### 4.4.1. Reminder-Recall Approaches

A message of a governmental source produced greater perceived trustworthiness and reduced vaccine hesitancy, among parents [45]. Messages from governmental sources are likely to produce better vaccination ratios, but more research on this topic is recommended.

Reminder-recall approaches or other communication interventions can positively change the behavior of vaccine hesitant parents. For instance, (i) parents with a negative post vaccination experience, (ii) parents who are more aware about risks vs. benefits of vaccination, and/or (iii) parents with less favorable opinion about vaccination schedule or about role of healthcare professionals may benefit from receiving messages or other reminder-recall approaches [45,47]. Particularly, parents’ beliefs and attitudes about health and the role of healthcare professionals (e.g., administration of vaccines) may negatively impact vaccine hesitancy [47].

Jarrett et al. (2015) reported that reminder-recall approaches (e.g., telephone call/letter) were:

(i) more predominant in higher income regions than in lower income ones (maybe because telephone call and/or postal communications are widely disseminated in high income countries).
(ii) associated to variable changes in vaccines uptake (maybe because more subjects are contacted through this type of intervention, which is likely to identify more causes of vaccine hesitancy), and
(iii) insufficient to control multiple causes of hesitancy (maybe because multifactorial causes of hesitance require multiple types of interventions to be controlled) [19].

#### 4.4.2. Tools to Evaluate Subject’s Vaccine Hesitancy

The development of a standardized set of questions about vaccine hesitancy may be useful, since questionnaires to evaluate parents’ vaccine hesitancy tend to be heterogeneous within studies [62,63]. In a developing country (Guatemala), participants presented significant constrains in understanding and using a Likert scale format to quantify vaccine hesitancy [46].

A tool to understand, evaluate, and monitor vaccine hesitancy in diverse global settings should be developed (e.g., Likert scales). Similar recommendations can be found in other systematic reviews: “methods to measure parental attitudes and beliefs about vaccination could be improved with validated and standardized yet flexible instruments” [62,63]. Further studies on this topic are recommended.

#### 4.4.3. Compulsory Vaccination

Compulsory vaccination may contribute to increase the number of vaccine hesitant individuals, with a potential reduction of the number of vaccinations [48]. However, variable impacts of compulsory vaccination on inoculations ratios are reported. Caregivers’ attitudes towards compulsory vaccination differed among immunization programs, e.g., between 53–97%. For instance, subjects were more resistant to get HPV vaccine [64].

### 4.5. Educational Strategies

#### 4.5.1. Educational Strategies for Health Professionals

Positively, the children of parents, who have consulted trained health professionals on vaccine hesitancy got more vaccines against influenza than the children of parents receiving usual care [52]. Tailored health behavior uptake models about vaccine hesitancy were followed. Among the adopted educational strategies were didactic or role-playing sessions [49,52].

As a consequence of presential and/or online training of health professionals, an improvement of knowledge, communication skills, comfort level, and ability to discuss vaccination or vaccine hesitancy were reported in diverse trials [13,49]. However, information-based training of health professionals may be less effective on vaccine uptake than communication tool-based training [19]. These findings strengthen the need of training healthcare professionals (e.g., physicians, nurses, or pharmacists) on vaccine hesitancy.

#### 4.5.2. Educational Strategies for Patients/Parents

Overall, training of parents about vaccination was related to positive outcomes [50,51], although changes were not reported in one of the selected studies [53]. Parents who received training and/or motivational interviews showed lower vaccine hesitancy and greater intention to vaccinate their children (e.g., motivational interviews at the maternity ward) than non-trained parents [50,51].

Further studies about the impact of tailored communication and training of parents and/or adolescents on vaccine hesitancy are recommended since research on this topic seems to be limited.

### 4.6. Study Limitations and Potential Biases of the Selected Studies

#### 4.6.1. Study Limitations of the Selected Studies

In general, methods were not fully reproductible, study biases were not discussed, and many topics were not addressed (e.g., the impact of different types of vaccines or communication of media on vaccine hesitancy). Additionally, the number of representative studies, multicentric research, prospective/longitudinal experiments, and investigations in developed and developing countries were limited. Studies were carried out in a restricted number of geographic areas: Germany (2 studies) [40,48], USA and India (1 study) [43], USA and Canada (1 study) [44], Guatemala and USA (1 study) [46], Canada (4 studies) [26,50,51,52], Malaysia (1 study) [47], and (25 studies) USA.

In the present systematic review: (i) the majority of the selected studies were from USA, (ii) studies simultaneously comprising strategies that address vaccine hesitancy and measuring the impact on vaccination ratios were limited, and (iii) study findings, settings, and target populations were heterogeneous. Similar findings were reported in the systematic review of Jarrett et al. (2015) [19].

#### 4.6.2. Potential Study Biases of the Selected Studies

Potential study biases of the selected studies (n = 35) were identified, as follows:

- Potential selection biases (“if the study population does not reflect a representative sample of the target population”) [65,66]: The selected studies were mainly non-representative (e.g., national representative studies were absent).
- Potential classification biases (“measurement or information bias, results from improper, inadequate, or ambiguous recording of individual factors”) [65,66]: The number and types of keywords, browsed databases, or inclusion and exclusion criteria were heterogeneous and, in some cases, limited.
- Potential confounding biases (i.e., “spurious association made between the outcome and a factor that is not itself causally related to the outcome and occurs if the factor is associated with a range of other characteristics that do increase the outcome risk”) [65,66]: Triangulations methodologies were not applied (e.g., just one tool or none tool was applied to characterize the level of subjects’ vaccine hesitancy) and in general, the number and type of collected sociodemographic data were limited.

### 4.7. Future Research Directions

Longitudinal, multicentric and representative studies about vaccine hesitancy of adults, children, caregivers, or health professionals are recommended in developed and developing countries, such as G7 economies (Japan, Germany, the UK, France, Italy, Canada) or in the Emerging Seven Markets (EM7) (China, India, Russia, Brazil, Indonesia, Mexico, and Turkey). Since 2017, around one-half of worldwide economic growth was attributable to the EM7. However, social inequalities, such as access and affordability of medical care or pharmaceuticals were prevalent in most of the EM7 in opposition to G7 economies [67]. Thus, the impact of health inequalities on patient’s vaccine hesitancy should be distinctly evaluated in different regions (e.g., development of validated tools to evaluate vaccine hesitancy in different populations/countries are required).

The exponential growth of chronic non-communicable diseases is challenging for the sustainability of health sector in both developed and developing countries [14,15,44,68], which reinforce the need of maintain high vaccination ratios in both regions. In addition, vaccine hesitancy and vaccination ratios need to be optimized at a global level, especially because of COVID-2019 pandemic.

Trials enrolling special populations are missing (e.g., geriatric, or oncologic patients, diabetics, hypertensives, cardiac patients, or health workers). For instance, healthcare workers remain suboptimal vaccinated against seasonal influenza, which is not an acceptable situation because of the collective goals to attain herd immunity (a “public good”), and, consequently, protecting the most vulnerable patients and/or health professionals [69]. Trials about vaccine hesitancy, regarding the vaccination against a specific disease are also suggested (e.g., SARS-Cov-2, herpes zoster, Measles–Mumps–Rubella, smallpox vaccine, etc.).

Ethical issues about vaccine hesitancy may be related to “a duty to patients (communitarian altruism), a duty to protect oneself, duty to one’s family, duty to colleagues and duty to society (solidarity)” [69]. Thus, research about ethical concerns is suggested. For instance, the development of studies about ethical topics may be especially relevant [14,15,44] during COVID-2019 pandemic, since non-immunized individuals may spread SARS-Cov-2 infection.

Studies about the impact of health literacy, social networks (e.g., Facebook and Instagram) or other media (e.g., TV and journals) on vaccine hesitancy are suggested. Communication studies seems to be especially relevant, since verbal or written interactions between health professionals and caregivers may improve vaccination ratios. Training about vaccine hesitancy (e.g., training of parents, residents, and physicians) or studies providing online information about vaccination (e.g., websites providing interactions with experts, apps, videos, or audio games) are also indicated. The design of studies comprising multiple interventions and triangulation methodologies are recommended since more resilient and hesitant subjects may require mixed interventions to get vaccinated.

Preferentially, research should be representative of a certain population/group and the impact of a certain intervention on vaccination ratios should be evaluated. Vaccine hesitancy seems to be a multifactorial and complex phenom. Research must be strongly theoretically grounded, documented, and reproductible (e.g., ideally study protocols should be public). Tailored research about vaccine hesitancy should be regularly carried out since the needs of a certain population is potentially variable during a certain period. Vaccine hesitancy must be continuously monitored in communities/populations, since the number of hesitant individuals is expectably variable.

Finally, the contact of citizens who have opted to no get a certain vaccine by health authorities is recommended to understand their motivations and to evaluate possible negative impacts (e.g., SARS-Cov-2 spread). This eventual individual contact must be strictly designed to respect ethical and deontological orientations and subjects’ individual opinions and rights (e.g., confidentiality of data).

### 4.8. Study Strengths

The 35 selected studies in the present systematic review accounted for at least 2023 health professionals or residents and 59,467 subjects. These participants were enrolled in intervention studies, which support a likely strength and relevance of study findings, i.e., all the findings were evidence-based. It is an advantage that no systematic reviews on the same topic and covering the same period (2015–2020) have been identified, which reinforces the importance and novelty of the present work.

Overall, key findings about “vaccine hesitancy” of trials enrolling patients and/or health professionals are summarized and discussed in the present systematic review (2015–2020), consequently making the available evidence more accessible to health professionals, researchers, decision makers, patients, and the public [70,71]. The main findings of the present work have been discussed considering other recent systematic reviews on related topics [19,54,55,56,57,58,59,60,61,62,63,64,65,66].

The present systematic review allows the identification of study gaps and the proposal of future research directions, namely about vaccine hesitant subjects (e.g., the study of vaccine hesitancy regarding vaccination against SARS-Cov-2).

One of the benefits of systematic reviews over meta-analysis is the higher chance of including and discussing more studies, since full statistical data are usually available in a limited number of works. For instance, a search about meta-analysis and vaccine hesitancy only retrieved a meta-analysis without time restrictions in PubMed (9 February 2021). This meta-analysis aimed to establish the summary effects of attitude, norms, and the perceived behavioral control on subjects’ vaccination intentions, with only 17 studies being selected. The theory of planned behavior was recognized and supported in terms of explaining vaccine hesitancy in this meta-analysis [72].

Finally, future research directions have been proposed, and potential study limitations discussed.

### 4.9. Potential Study Limitations of the Present Systematic Review

A specific set of keywords about a certain topic is required in systematic reviews. The PRISMA criteria/checklist for systematic reviews particularly requires that searches: “present full electronic search strategy for at least one database, including any limits used, such that it could be repeated” [17,73]. This requirement was followed in the present work.

The limited number of selected keywords may have restricted the number of selected studies (n = 35). For instance, more search strategies were applied in Lawes-Wickwar et al. (2021) (“vaccine”, “vaccines”, and “vaccination”) [74] and in Jarrett et al. (2015) (“strateg *”, “intervent *”, “campaign”, “evaluation”, “approach”, or “program *”) [19]. Thus, an additional systematic review using more keywords is recommended. Among the suggested keywords are immunization, vaccine confidence, experimental studies, and distrust.

## 5. Conclusions

The number of identified trials, research topics, multicentric and/or longitudinal/prospective studies related to vaccine hesitancy were limited in the last five years (2015–2020). Studies were heterogeneously designed, many topics were not researched (e.g., vaccine hesitancy regarding different types of vaccines), methods were not fully reproducible, authors had not endorsed potential study biases and at least two studies did not produce positive outcomes [40,53]. Additionally, these trials were non-representative (i.e., national representative studies are lacking) and, in some cases, studies involved a limited number of participants. Thus, study findings may not be representative.

It seems the control of pain and distress in vaccinated children contributes towards the limiting of parents’ vaccine hesitancy (e.g., topical lidocaine and/or non-pharmacological measures may be recommended in pediatric vaccination). The provision of online or electronic information (e.g., trough virtual reality, social websites of experts, apps, or internet-based platforms) is likely to reduce vaccine hesitancy. Intervention-based communication (e.g., written or oral) supporting HPV, influenza, or diphtheria tetanus toxoid and pertussis vaccination and an explanation of the potential complications of these diseases seem essential to combat vaccine hesitancy. Minority and disfavored groups may benefit from tailored vaccination strategies to increase immunization (e.g., close collaborations between health professionals and less favored communities).

Tailored educational strategies (e.g., motivational interviews and/or online training for parents, healthcare professionals or residents) about vaccine hesitancy and/or explaining the complications of the related diseases and/or benefits seem to reduce vaccine hesitancy. A higher vaccination hesitancy was found in parents who were more aware of the risks than the benefits of vaccination, parents with a negative post vaccination experience, parents with a less favorable opinion about vaccination schedules or the role of healthcare professionals, parents with a fear of needles, or the imposition of compulsory vaccination. A universal tool to evaluate and monitor subjects’ vaccine hesitancy seems to be lacking in both developed and developing countries.

In general, the conclusions of the present systematic review are aligned with those of Jarret et al. (2015), although studies about targeting multiple audiences (e.g., involvement of religious or traditional leaders, social media, or mass media) were not identified in the present systematic review. These studies were mainly identified in developing countries. According to the findings of the systematic review of Jarrett et al. about vaccine hesitancy (2007 to 2013), dialogue-based approaches through targeting multiple audiences were more likely to produce positive outcomes. Vaccine hesitancy was recognized as a complex issue. The selected studies were classified as highly heterogeneous about their design and outcomes; consequently, it was not possible to define more general study conclusions [19].

## Figures and Tables

**Table 1:** Main findings of the 35 selected studies

Author(s), Year, Country, DataBase	Objective(s)	No. of Participants	Methods and Results	Conclusions
**Children/pediatric**				
1. (Abukhaled et al., 2020) [11] USA, Bon	To alleviate parental concern about pain and to facilitate infant immunization.	30 pediatric nurses; 100 participants	Methods: A nonpharmacological intervention (breastfeeding or 24% sucrose solution: infants up to 6 months). Results: a significant reduction of parental concern about vaccine pain (pre- vs. postintervention; p = 0.035).	Nonpharmacological methods may relieve infants’ pain undergoing vaccination, improve vaccination rates and reduce parental concern about pain.
2. (Opel et al., 2019) [7] USA PubMed	To evaluate the effect of vaccine hesitancy screening on childhood vaccine uptake.	156 parents (65 intervention)	Methods: Parents received the Parent Attitudes About Childhood Vaccines Survey (PACV). Placebo survey items were purposively included in PACV. Intervention: providers received a summary of PACV responses. Control: Results of placebo items were provided. Outcome: Immunization status of children (8 months of age) expressed as % of days under-immunized. Results: outcomes between groups were not statistically significant.	Parents’ vaccine hesitancy was not significantly associated with % of days under-immunized.
3. (Henrikson et al., 2017) [26] USA PubMed	To evaluate how parental vaccine hesitancy changes with the age of children.	237 mothers	Methods: Intervention (providers received training) and control (no training). Results: Both groups presented similar proportions of vaccine hesitant parents (baby’s birth and at 6 and 24 months). The proportion of mothers who were vaccine hesitant significantly decreased (p = 0.01) from child’s birth to age 24 months.	Vaccine hesitancy was variable, with a peak around childbirth and a likely remit as parents’ experience with vaccines increase.
4. (Philpott et al., 2017) [27] USA PubMed	To examine clinician’s attitudes, beliefs, and behavioral intentions about discussing evidence and eliciting values.	132 medical students and pediatric residents	Methods: Participants were required to read three scenarios. One scenario: a parent of a one-year-old children was hesitant about vaccination. Administration of an opinion questionnaire. Results: Health professionals considered the conversation about vaccine hesitancy less relevant, perceived parents as difficult, and had lower respect for parents’ views (p < 0.0001).	Attitudes of clinicians seems to impact conversations with vaccine hesitant patients.
5. (Taddio et al., 2017) [28] Toronto, ON, Canada PubMed	To compare the effectiveness of additive pain interventions during vaccine injections in the first year of life.	352 infants	Methods: groups: (i) placebo (control); (ii) parent-directed video education about infant soothing; (iii) video plus oral sucrose or (iv) video, oral sucrose, and topical lidocaine (vaccine injections at 2, 4, 6 and 12 months). Injection techniques to minimize pain (all groups). The Modified Behavioral Pain Scale was applied to evaluate pain distress at pre injection, vaccine injection (needle), and 1 min post injection (recovery). Results: Needle pain significantly differed among groups (p = 0.003) and across ages (p < 0.001). Consistent analgesia was only achieved with topical lidocaine.	Topical lidocaine during vaccination seems to control pain in the first year of life.
Online or electronic information				
6. (Nowak et al., 2020) [8] USA Cochrane Library	To evaluate if the administration of vaccine information statements (VIS) plus an immersive virtual reality (VR), short video or electronic pamphlet story conveying community immunity benefits of influenza vaccination ameliorate (or not) participants’ influenza-related perceptions and influenza vaccination-related beliefs, confidence, and intentions.	171 participants (Flu vaccine avoidant).	Methods: Intervention groups: (i) VR + VIS; (ii) video + VIS; and (iii) e-pamphlet + VIS. Control: an influenza VIS-only. VR: Participants wore a head-mounted display unit and video game controllers to experience a five-minute VR story. Video: The same content of the VR condition. e-pamphlet: the text and static images of the video were viewed in a tablet. Results: VR produced a stronger perception of presence in the story, increased participants’ concerns about transmitting influenza to others and boosted vaccination intention. Participants´ concerns about transmitting influenza encouraged vaccination.	VR increased participants’ understanding of key immunization concepts through promoting the sense of presence in story. VR may be useful to address the problem of vaccination hesitancy.
7. (Shoup et al., 2019) [29] USA PubMed	To evaluate and compare the interactive content of an expert moderated vaccine social media (VSM) website, which was purposively developed for parents of children 24 months of age or younger vs. publicly available websites (PAW).	542 mothers (and partners).	Methods: two groups: PAW and VSM. Vaccine hesitancy was accessed with Parent Attitudes and Childhood Vaccines survey. Results: Tone, vaccine stance, and accuracy of information were significantly better in VSM than PAW (p <0.05). Participants’ interactions: ask questions to vaccine experts (n = 36; 59%), chat sessions (n = 15; 25%), blogs (n = 7; 11%), and discussion boards (n = 3; 5%).	VSM offered a suitable platform for parents to collect accurate vaccine information, express vaccine concerns and ask questions to experts. Regarding VSM consultation, participants were less likely to post inaccurate information. PAW tended to be contentious, presented a negative stance toward vaccines and comprised inaccurate information.
8. (Salmon et al., 2019) [30] USA Cochrane Library	To evaluate self-reported information needs (pre-and post-videos), among pregnant woman using a MomsTalkShots (an app) and to evaluate app usability.	1103 pregnant woman.	Methods: pre- and post-videos (app). Videos: animations to communicate attracting and interesting messages about vaccination. Participants’ sociodemographic data and vaccine attitudes, beliefs, and intentions were collected with a questionnaire. Results: MomsTalkShots (classification): helpful (95%), trustworthy (94%), interesting (97%), and clear to understand (99%). Similar classifications were obtained for vaccine hesitant woman. 72% of woman who mentioned not having enough vaccine information pre-videos, subsequently reported enough information (post-videos).	The developed app was well-received among pregnant women, even among vaccine hesitant ones.
9. (Daley et al., 2018) [31] USA PubMed	To assess the impact of an internet-based platform on parent’s vaccine related attitudes.	945	Methods: A questionnaire about parent’s vaccine-related attitudes (at baseline and when their child was aged 3–5 and 12–15 months). Groups: (i) a website with vaccine information and social media components; (ii) a website with vaccine information only or (iii) usual care. Outcome: Changes in parent’s vaccine attitudes. Results: Vaccine hesitant parents showed significant improvements on vaccination benefits. Parent’s concerns about vaccination risks were significantly reduced.	An internet-based intervention positively improved the attitudes of vaccine hesitant parents.
10. (Glanz et al., 2017) [32] USA PubMed	To determine if a Web-based, social media intervention increases early childhood immunization.	888 pregnant women	Methods: Groups: (i) a Website with vaccine information and interactive social media (VSM), (ii) a Website with vaccine information (VI), or (iii) usual care (UC). Outcome: Number of days under-vaccinated (from birth to age of 200 days). Results: The mean ranks of the number of days under-vaccinated were significantly lower in the VSM arm vs. UC (p = 0.02). Proportions of infants up to date at age of 200 days: 92.5 (VSM), 91.3 (VI), and 86.6 (UC).	Web-based vaccine information and social media applications may positively influence parental vaccine behaviors.
Vaccination against a specific disease: Human papillomavirus infection				
11. (Khodadadi et al., 2020) [9] USA Cochrane Library	To evaluate HPV vaccine hesitancy, among Latina immigrant mothers of daughters (9–12 years).	317 Latina immigrant mothers	Methods: A survey was administered at baseline (participant’s sociodemographic data, knowledge and perceived risk of cervical cancer/HPV infection, self-efficacy, and intention to vaccinate their unvaccinated daughters). Mothers’ vaccine hesitancy was quantified. Results: 35.3% of vaccine hesitant mothers. Multivariable model: explanatory variables of vaccine hesitancy (daughter’s health insurance status; HPV awareness; perceived risk of HPV infection; perceived self-risk of cervical cancer; and a self-efficacy score of ability to complete the HPV vaccination series).	Latina immigrant mothers may not feel motivated to vaccinate their daughters after receiving physicians´ recommendations. Mother’s awareness on other HPVvaccination topics, such as HPV and cervical cancer should be improved.
12. (Reno et al., 2020) [33] USA Cochrane Library	To evaluate the impact of a multi-component communication-based intervention about HPV vaccination (11–12 years children) on vaccination rates.	149 (baseline) and 187 (post-intervention) providers	Methods: Baseline (n = 75 control and intervention n = 74) and post-intervention (control n = 108 and intervention n = 79). Intervention group: a motivational interviewing and a fact sheet with vaccine hesitant parents (communication components). Results: Providers reported higher perceived levels of parental HPV vaccine acceptance in intervention than control. Communication components improved HPV vaccination rates.	A multi-component communication-based intervention may contribute to improve the perceived level of parental HPV vaccine acceptance and vaccination rates.
13. (Panozzo et al., 2020) [34] USA Cochrane Library	To evaluate if supplementing tailored messages that address parental concerns about human papillomavirus (HPV), improve HPV vaccination intent among mothers.	259 (general video); 249 (top concern video); 237 (al concerns videos)	Methods: Mothers (HPV vaccine hesitant) of 11 to 12 years children. Groups: (1) “control” (only the bundled recommendation video); (2) “top concern” (control video plus a top concern video); or (3) “all concerns” (control video plus videos addressing all concerns). Results: The mean intent to vaccinate postintervention ranged from 3.5 (control)–4.2 (all-concerns group) (p = 0.01). The mean strength of the concerns declined pre- to postintervention by 0.1 in control group to 0.7 in all concern’s group (p < 0.001). The main concerns of hesitant mothers remained high in postintervention.	Tailored messages addressing all concerns improved HPV vaccination intention and reduced the strength of the main concerns of vaccine hesitant mothers.
14. (Dempsey et al., 2019) [35] USA PubMed	To evaluate the impact of a provider communication intervention on improving adolescent HPV vaccination.	777 parents of adolescents	Methods: Eight control and 8 intervention clinics. Intervention: provider communication aiming at improving adolescent HPV vaccination. Results: A ‘very strong’ recommendation about HPV vaccination produced a greater perceived urgency for getting vaccinated, greater trust in the received information, decreased vaccine hesitancy, and increased vaccine receipt. Similar findings were found for presumptive recommendations.	A provider very strong HPV vaccine recommendation or presumptive recommendation were related to improved parent attitudes and acceptance of this vaccine.
15. (Kornides et al., 2019) [36] USA Cochrane Library	To evaluate if a presumptive, bundled recommendation with tailored message addressing one vs. all parental concerns improves HPV vaccination intent among mothers of girls versus boys.	762	Methods: Mothers who have no intention of getting vaccinated their child (11–14year-old) against HPV (27 states). Groups: a) presumptive, bundled recommendation video (“control”); b) control + video addressing the top concern; or c) control + ≥1 video addressing all concerns. Outcome (mean): HPV vaccination intent (1 = extremely unlikely and 10 = extremely likely). Results: the mean intent to vaccinate between the intervention group (all concerns) and control was statistically significant (mothers of boys post intervention) and the mean intent to vaccinate between both intervention groups (top concern or all concerns) and control was statistically significant (mother of girls post intervention).	Tailored messages addressing all concerns positively improved mother’s (boys and girls) vaccination intent. Videos addressing top concerns improved mothers´ intention of vaccinate against HPV their daughters.
16. (Reno et al., 2018) [37] USA PubMed	To analyze the impact of motivational interviews on HPV vaccination rates.	46 providers	Methods: Eight intervention and control clinics. Intervention: health professionals (e.g., nurse practitioners or physician assistants) conducted motivational interviews. Results: vaccination rates were better in intervention than control.	Motivational interviews played a central role in improving HPV vaccine acceptance.
17. (Henrikson et al., 2018) [38] USA PubMed	To evaluate the impact of health system-based outreach and reminders on HPV vaccine series initiation and completion.	1805 parents of 10–12 year children.	Methods: Groups: (1) first an outreach letter and brochure recommending HPV vaccination was offered followed by automated HPV vaccine reminders (intervention) or (2) usual care (control). Results: HPV vaccine initiation was higher in the intervention than control at 120 days after randomization (23.6% and 18.8%, p = 0.04) and at study completion (10.3% vs 6.8%, p = 0.04).	Written information and reminders are likely to improve HPV vaccination of 10–12-year children.
18. (O’Leary et al., 2017) [39] USA Cochrane Library	To evaluate the impact of a communication intervention on adolescent HPV vaccine uptake.	188 medical providers and 43,132 adolescents (11–17 years old)	Methods: Control vs. a five-component intervention (1) HPV fact library, (2) an education website, (3) HPV-related images, (4) a decision aid on HPV vaccine, and (5) 2 h of in-person communication training on using a presumptive vaccine recommendation followed by a motivational interview for vaccine hesitant parents. Outcome: Differences between control and intervention practices at initiation (≥1 dose), and completion (≥3 doses). Results: the intervention group registered a significantly higher odds of HPV series initiation (≥1 dose) and completion (≥3 doses) over time than control.	The 5-components intervention significantly improved HPV vaccine series initiation and completion among adolescents.
19. (Baxter et al., 2017) [12] USA PubMed	To examine the relationship between preschool vaccine history, parent and preadolescent needle fear, and subsequent compliance with optional vaccines.	120 preadolescents	Methods: quantification of needle anxiety: parents and 10–12-year-old preadolescents (100 mm visual analogue scale). Collected information: needle anxiety (previous vaccination), number of vaccinations between the ages of four and six years (total and same day), and subsequent initiation of HPV vaccine. Results: 12.5% (15) of parents reported anxiety about vaccination. Parent and child anxiety were weakly correlated (r = 0.15). Eight children (26.67%) in the upper fear quartile began their HPV series compared to 14 (48.28%) in the lower quartile (OR 2.57, p = 0.0889, 95% CI 0.864–7.621).	Preadolescent fear of needles was a stronger predictor of non-compliance with HPV vaccine than parent vaccine anxiety. Same-day preschool injections (between 4–6 years) increased the chance of a child show fear of needles five years later.
Vaccination against a specific disease: influenza vaccination				
20. (Nekrasova et al., 2020) [10] USA Cochrane Library	To assess vaccine hesitancy, knowledge, attitudes, and beliefs among caregivers of children who received the first of two doses of flu vaccine.	256 participants	Methods: 36 primary care practices (24 states). A reminder (text-message) of the second dose of influenza vaccine was send. Intervention: telephone survey to collect demographic information; the Parent Attitudes About Childhood Vaccines Survey Tool was administered and caregivers were questioned about influenza vaccine and disease. Results: 11.7% of caregivers were moderate/high vaccine hesitant. Many participants manifested misperceptions about influenza and flu vaccine.	Some caregivers may remain vaccine hesitant or maintain inaccurate influenza beliefs even after the administration of the first dose of flu vaccine.
21. (Giese et al., 2020) [40] Bon, Germany	To tests how groups of individuals with positive and negative attitudes towards flu vaccination attend to and convey information online.	208 (negative) and 221 (positive attitudes towards flu vaccination)	Methods: Three links were tested. Outcomes: (i) what type of information about flu vaccination was conveyed to the subsequent link, (ii) how subjects´ perceptions about flu-vaccination were altered by incoming messages, and (iii) how participants perceived incoming information. Results: (i) participants selectively conveyed attitude-consistent information, (ii) participants were reluctant to alter their perceptions about flu-vaccination in response to messages, and (iii) participants consistently evaluated the incoming information in line with their prior attitudes.	Online messages were not enough relevant to change participant’s perceptions and attitudes towards flu vaccination.
22. (Real et al., 2017) [41] USA PubMed	To evaluate the impact of physician-patient communication on parent’s refusal of influenza vaccination (children aged 6 to 59 months).	45 Residents	Methods: intervention (n = 24 residents) and control (n = 21 residents). Intervention: Three virtual reality simulations. Residents were required to instruct avatars expressing vaccine hesitancy. Outcome: impact of the curriculum on refusal rates of influenza vaccine between the intervention and control groups (3 months after the virtual reality curriculum). Results: the refusal of influenza vaccine in intervention was lower than control (27.8% vs 37.1%; p = 0.03) (post curriculum period).	A virtual reality curriculum may be an effective tool for the training of medical residents (communication skills).
Vaccination against a specific disease: diphtheria tetanus toxoid and pertussis3				
23. (O’Leary et al., 2019) [42] USA Cochrane Library	To evaluate the impact of an online vaccine resource on the uptake of the vaccines against tetanus, diphtheria, and acellular pertussis (Tdap) and influenza vaccines among pregnant women.	289 (influenza) and 173 (Tdap) pregnant women	Methods: Groups: (i) a website with vaccine information and interactive social media components (VSM), (ii) a website with vaccine information only (VI), or (iii) usual care only (UC). Outcomes: vaccination rates (Tdap and influenza). Results: influenza vaccine uptake was significantly higher in both intervention arms (57% VSM and 55% VI) than the usual care (36%). Tdap: statistically significant differences between arms (71% VSM, 69% VI, and 68% UC) were not found.	Web-based vaccination information may positively influence influenza vaccine uptake, among pregnant women.
24. (Nagar et al. 2018) [43] USA and India PubMed	To compare the timely DTP3 (diphtheria tetanus toxoid and pertussis3) adherence amongst three study arms and to compare mothers’ survey responses about field communication.	198 mothers	Methods: 96 village health camps in India. Arms: (i) Near Field Communication (NFC) sticker (control group), (ii) NFC necklace, and (iii) NFC necklace with voice call reminder (local dialect). Outcomes: to compare the timely DTP3 adherence amongst all three study arms (primary outcome) and to compare mother’s survey responses about symbolic necklaces (secondary outcome: field communication). Results: NFC necklace and the necklace plus voice call reminders (local dialect) not directly promoted an increase in infant DTP3 timeliness immunization (primary outcome). The provision of a necklace generated a significant community discussion (p = 0.0118), a strong satisfaction (p < 0.0001) and an increased visibility within families (p < 0.00001) (secondary outcome).	Symbolic necklaces may constitute an assistive tool in immunization campaigns.
Vaccination against a specific disease: COVID-2019				
25. (Bogart et al., 2020) [44] USA and Canada PubMed	To examine associations between medical mistrust on COVID-2019 vaccine and treatment and patients´ adherence to antiretroviral therapy (ART).	101 HIV-positive Black Americans	Methods: a telephone interview to evaluate: COVID-2019 mistrust), COVID-2019 vaccine and treatment hesitancy, and trust in information about COVID-2019. Patient’s adherence to antiretroviral therapy was quantified. Results: over half of the participants manifested at least one COVID-2019 vaccine or treatment hesitancy belief. A lower antiretroviral therapy adherence was related to more negative COVID-2019 behaviors (p = 0.02).	Tailored strategies should be implemented to promote COVID-2019 vaccination and treatment, to overcome mistrust and to prevent health inequalities in less favored communities.
Miscellaneous: extrinsic factors				
26. (Xu et al., 2020) [45] USA Cochrane Library	To evaluate if messages from a governmental source improve perceived source credibility, increment parent intentions to vaccinate their children, and/or reduce vaccine hesitancy.	1000 (t1) and 800 (t2)	Methods: Tailored messages were sent by the Centers for Disease Control and Prevention to convey the expertise, trustworthiness, or caring/goodwill. Study hypothesis: to evaluate if the tailored messages improve perceived source credibility, increment parent intentions to vaccinate their children, and/or reduce vaccine hesitancy. Results: Overall, study hypothesis were not demonstrated, although some relevant findings have emerged from this study.	Tailored messages to communicate source expertise produced greater perceived caring/goodwill among parents who were vaccine hesitant at baseline.
27. (Domec et al., 2018) [46] Guatemala and USA PubMed	To identify and compare parents´ vaccine hesitancy in rural and urban Guatemala.	720	Methods: Clinics (rural and urban Guatemala). Parents of infants (6 weeks to 6 months) completed a questionnaire on vaccine hesitancy (Likert scale). Results: 40.8% think “most parents like them have their children vaccinated with all the recommended vaccines”. Participants had difficulty in understanding and using the Likert scale. Factor analysis showed a two-factor structure within the vaccine hesitancy Likert scale.	A significant proportion of vaccine hesitant parents was identified, with these parents constituting a very heterogeneous group. Thus, the development of a survey tool to identify vaccine hesitant parents was complex.
28. (Karimah et al., 2017) [47] Malaysia Cochrane Library	To identify variables with influence on vaccine hesitance among parents from Kuantan, Pahang.	155 parents	Methods: administration of a questionnaire. Results: in the logistic regression analysis, parents’ beliefs and attitude about health and prevention and/or the role of healthcare professional were significantly associated with vaccine hesitancy. The most explanatory variables of vaccine hesitancy: post vaccination experience (95.5%); vaccination schedule (60.5%), role of healthcare professional, awareness (56.1%) and risks and benefits of vaccination (51.6%).	The role of healthcare professionals and parent’s beliefs and attitudes about health may negatively impact vaccine hesitancy.
29. (Betsch and Böhm, 2016) [48] Germany PubMed	To assess the effect of partial compulsory vaccination on the subsequent uptake of other voluntary vaccines.	297	Methods: Two sequential vaccination decisions were simulated (online experiment). Groups: (i) compulsory vaccination (n = 144) (intervention) and (ii) voluntary vaccination (n = 153) (control). Voluntary vaccination was the second decision for both groups. Results: The second voluntary vaccination was reduced by 39% in the intervention group.	Compulsory vaccination may negatively impact vaccination rates.
Educational Strategies				
30. (Pahud et al., 2020) [13] USA PubMed	To develop and evaluate an online curriculum on vaccine education for pediatrics and family medicine residents.	1444 residents (734 intervention, 710 control).	Methods: Four online modules and an in-person training guide (intervention). Standard vaccine education (control sites). Pre-intervention and post-intervention surveys were emailed to residents (both groups). Primary outcomes: “Vaccine knowledge,” “vaccine attitudes/hesitancy,” and “self-confidence”. Results: Intervention residents (online curriculum) showed greater self-confidence and ability to discuss vaccines with parents/patients than control (p < 0.03). Vaccine hesitancy was higher for family medicine residents (23%) than pediatric residents (10%).	The developed online curriculum seems to be an effective model of immunization education for residents.
31. (Norton et al., 2019) [49] USA Cochrane Library	To evaluate if the residents attending a curriculum on vaccine hesitancy demonstrate improved knowledge, comfort, and skills.	70 residents	Methods: Control and intervention (35 residents). Curriculum design framework: didactic sessions per pediatric vaccine and interactive role-playing sessions on communication skills. Residents of both groups were required to assess a standardized patient (pre and posttests). Results: Pretests scores were not statistically significant different between both groups. The standardized patient was better scored in intervention than control (78% vs. 52%, p = 0.00). Post curriculum: statistically significant better scores were found for residents´ knowledge (47–66%) and comfort level (2.9/5–3.76/5) in intervention than control.	The developed curriculum produced improvements on resident’s vaccine knowledge, communication skills and comfort level
32. (Gagneur et al., 2019) [50] Quebec, Canada, PubMed	To assess vaccination intention and vaccine hesitancy among parents who received an individual motivational interview on infant immunization during post-partum stay at a maternity ward.	1223 parents	Methods: Post-partum stay at a maternity ward. Intervention: motivational interview (pre/post-intervention in four maternities). Parents filled pre- and post-intervention questionnaire on vaccine intention and vaccine hesitancy. Results: Parents´ vaccine intention increased by 12% (78% vs 90%, p < 0.0001), and parents´ vaccine hesitancy reduced by 40% (27% vs 16%; p < 0.0001) post-intervention.	A parents’ motivational interview on immunization produced positive outputs: a lower vaccine hesitancy and a greater intention to vaccinate their infant at 2 months of age.
33. (Gagneur et al., 2018) [51] Quebec, Canada, PubMed	To assess the impact of an educational strategy about vaccination promotion, among parents who delivered at the maternity ward.	Parents of 1140 (intervention) and 1249 newborns (control)	Methods: An individual educational information session about immunization was administered at maternity ward (intervention). Control: usual care. Results: vaccine coverage significantly increased in intervention than control (p < 0.05): 3.2, 4.9, and 7.3%, respectively at 3, 5, and 7 months.	Motivational interviews may constitute effective tools to overcome parents´ vaccine hesitancy.
34. (Fisher et al., 2018) [52] Ontario & Quebec, Canada Cochrane Library	To evaluate the impact of an Information—Motivation—Behavioral Skills model (IMB)-based, accredited, online Continuing Medical Education (CME) program on seasonal influenza vaccination.	68 healthcare professionals	Methods: 33 healthcare professionals in CME/intervention (292 visits) and 35 healthcare professionals in control (336 visits). Children 6–23 months. Intervention: the CME comprised information/training about tailored health behavior. Results: parents in the CME group were around ~30% more likely to agree to immunize their child with influenza vaccine than parents in control (p = 0.007).	Medical education programs may contribute to a better influenza immunization of children.
35. (Henrikson et al., 2015) [53] USA PubMed	To test a communication intervention, which was designed to reduce mother’s vaccine hesitancy and to increase physician confidence in communicating about vaccines.	347 mothers	Methods: 30 mothers (intervention) and 26 mothers (control) (56 clinics). Intervention: physicians received a targeted communication training. Mothers and physicians were evaluated at baseline and 6 months. Vaccine hesitancy was quantified with the Parental Attitudes on Childhood Vaccines score. Results: Both maternal vaccine hesitancy and physician self-efficacy in communicating about vaccines with parents were not improved by the communication training.	The communication intervention not produced positive outcomes.

## Data Availability

The data presented in this study are available in the article.
